# Mutually exclusive dendritic arbors in *C*. *elegans* neurons share a common architecture and convergent molecular cues

**DOI:** 10.1371/journal.pgen.1009029

**Published:** 2020-09-30

**Authors:** Rebecca J. Androwski, Nadeem Asad, Janet G. Wood, Allison Hofer, Steven Locke, Cassandra M. Smith, Becky Rose, Nathan E. Schroeder

**Affiliations:** 1 Neuroscience Program, University of Illinois at Urbana-Champaign, Urbana, Illinois, United States of America; 2 Department of Crop Sciences, University of Illinois at Urbana-Champaign, Urbana, Illinois, United States of America; University of California San Diego, UNITED STATES

## Abstract

Stress-induced changes to the dendritic architecture of neurons have been demonstrated in numerous mammalian and invertebrate systems. Remodeling of dendrites varies tremendously among neuron types. During the stress-induced dauer stage of *Caenorhabditis elegans*, the IL2 neurons arborize to cover the anterior body wall. In contrast, the FLP neurons arborize to cover an identical receptive field during reproductive development. Using time-course imaging, we show that branching between these two neuron types is highly coordinated. Furthermore, we find that the IL2 and FLP arbors have a similar dendritic architecture and use an identical downstream effector complex to control branching; however, regulation of this complex differs between stress-induced IL2 branching and FLP branching during reproductive development. We demonstrate that the unfolded protein response (UPR) sensor IRE-1, required for localization of the complex in FLP branching, is dispensable for IL2 branching at standard cultivation temperatures. Exposure of *ire-1* mutants to elevated temperatures results in defective IL2 branching, thereby demonstrating a previously unknown genotype by environment interaction within the UPR. We find that the FOXO homolog, DAF-16, is required cell-autonomously to control arborization during stress-induced arborization. Likewise, several aspects of the dauer formation pathway are necessary for the neuron to remodel, including the phosphatase PTEN/DAF-18 and Cytochrome P450/DAF-9. Finally, we find that the TOR associated protein, RAPTOR/DAF-15 regulates mutually exclusive branching of the IL2 and FLP dendrites. DAF-15 promotes IL2 branching during dauer and inhibits precocious FLP growth. Together, our results shed light on molecular processes that regulate stress-mediated remodeling of dendrites across neuron classes.

## Author summary

Neurons have processes called dendrites that receive information. Dendrites are often elaborately shaped and highly branched. Adverse stress in the mammalian brain can reduce dendritic branching in some neuron types, while increasing it in others. How stress can cause opposite branching responses in different neurons is unclear. The transparent nematode *C*. *elegans* has a defined and relatively simple nervous system amenable for study. The head of *C*. *elegans* contains two classes of neurons with highly arborized dendritic arbors. The FLP neurons form dendritic arbors as part of healthy reproductive development. In contrast, the IL2 neurons undergo dendritic arborization only during a stress-induced developmental stage. When fully arborized, FLP and IL2 dendrites occupy the same area, although during distinct developmental stages. Using forward and reverse genetics, we find that branching in both neuron types is controlled by a similar protein complex, but that regulation of this complex differs between cell types. Our data demonstrate how molecular cues drive the transition between stressed and unstressed states in the nervous system.

## Introduction

Dendrite morphology is crucial for efficient neural signaling. Recent years have seen increased attention given to the molecular basis of dendritic arborization using both cell-culture studies and model organisms. Experience-driven remodeling of a dendrite can contribute to both the size and complexity of dendritic arbors in a diverse range of species. Depending on neuron class, these changes can include both increasing and decreasing dendritic volume [1–3]. For example, dendrites in the basal lateral amygdala undergo hypertrophic growth following chronic stress [4], whereas those of the hippocampus atrophy [5]. However, little is known about the molecular mechanisms that cause differential arborization in different neuron classes during normal physiological conditions versus stress-exposed conditions. Similarly, what, if any, coordination occurs among distinct neuron types is unknown.

**Fig 1:**
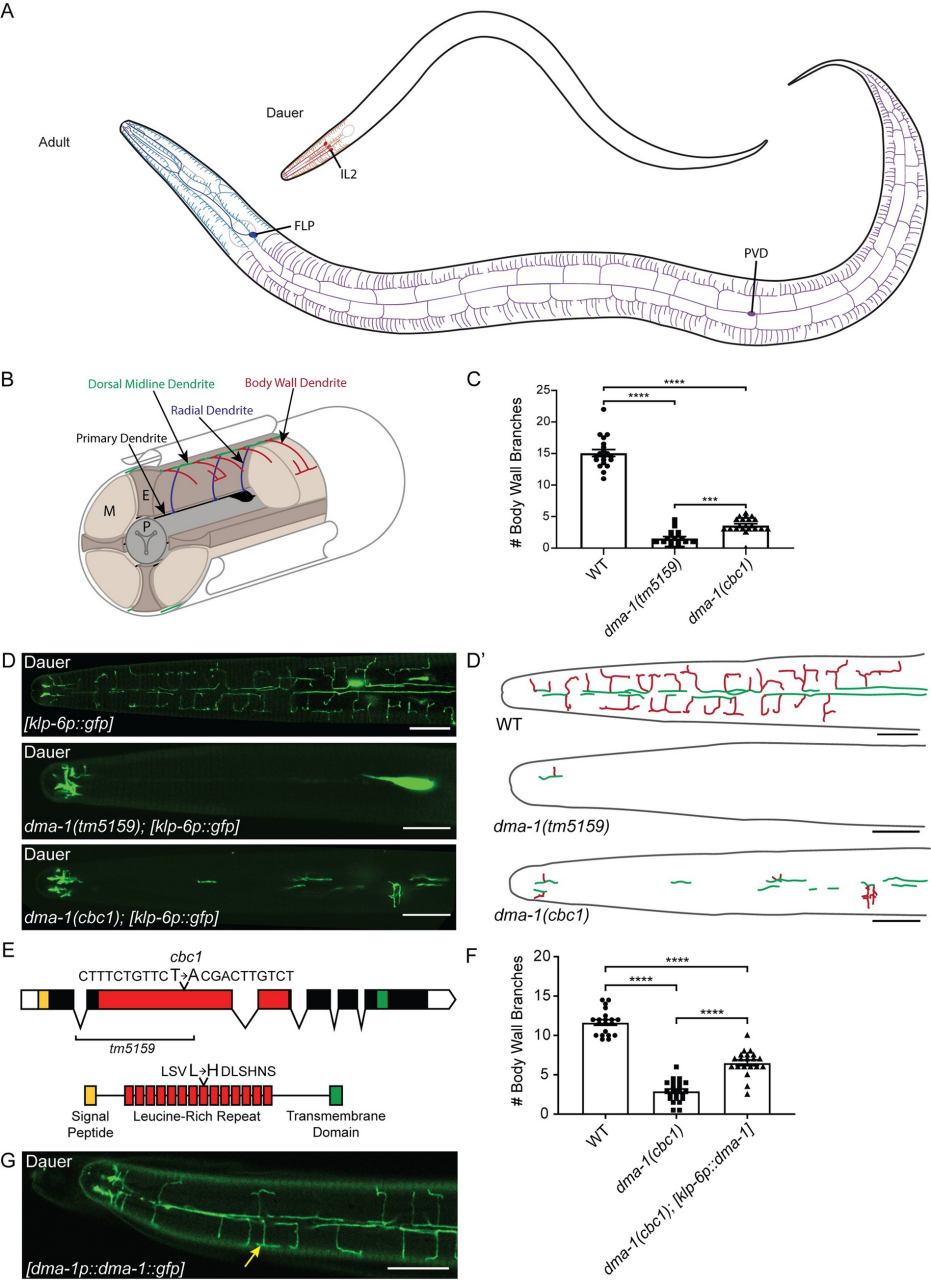
DMA-1 is required in the IL2s for arbor formation. (A) There are three classes of highly arborized neurons in *C*. *elegans*: IL2, FLP, and PVD. The IL2s (red) arborize strictly during the dauer stage where their arbors are restricted to the head of the nematode. The FLPs (blue) and PVDs (purple) form arbors during late L4-adult and together cover the head and body, respectively. (B) A schematic cross-section of the IL2 branching pattern, shown in ¾ view. The IL2 cell body and primary dendrite (established during embryonic development) are black. Subsequent dendritic growth occurs during dauer formation leading to branches from the primary dendrites towards the ventral and dorsal midlines in a radial arrangement (blue), branches along the midlines (green), and body wall branches extending from the midlines (red). P = pharynx, E = epidermis, M = muscle. (C) Quantification of the number of IL2 branches along the body wall in wild-type (n = 20), *dma-1(tm5159)* (n = 21), and *dma-1(cbc1)* (n = 21). (D) Z-projection confocal micrographs of IL2 body wall branches in dauers. *dma-1(cbc1)* mutants have severe defects in IL2 arborization. The deletion allele, *dma-1(tm5159)*, has a greater reduction of branching than the *dma-1(cbc1)* allele. The IL2 neurons are labeled with *klp-6p*::*gfp*. (D’) Schematic drawings of IL2 arbors traced from the accompanying micrographs illustrating the dorsal midline (green) and body wall branches (red). (E) Gene schematic of *dma-1* (top). *dma-1(cbc1)* is a point mutation that results in a T to A transversion within the leucine-rich repeat (red) region. The previously described deletion allele *dma-1(tm5159)* removes much of the LRR domain (9). Protein domain schematic (bottom) highlights the leucine to histidine mutation in *dma-1(cbc1)* within the eighth leucine-rich repeat. (F) Quantification of the number of IL2 branches along the body wall during dauer in wild-type (n = 19), *dma-1(cbc1)* (n = 18), and *dma-1; klp-6p*::*dma-1* (n = 20). Expression of *dma-1* in the IL2 neurons using the *klp-6* promoter partially rescues the *dma-1* mutant branching phenotype. (G) Z-projection confocal micrograph of a dauer expressing *dma-1*::*gfp* under the *dma-1* promoter. *dma-1p*::*dma-1*::*gfp* localizes to the IL2 dendrites (yellow arrow) during dauer. This image substack includes the dendrites located at the body wall. For IL2 body wall branch quantification (C and F), an ANOVA followed by Tukey’s test for multiple comparisons was used to determine significance, ***p ≤0.001 and ****p ≤0.0001. Error bars, standard error of the mean. Scale bars, 10 μm.

The nematode C*aenorhabditis elegans* is one of several leading models for studying dendritic arborization [3,6–8]. Its transparent body, well-described nervous system, and abundant genetic tools available for its study comprise a robust system for understanding the molecular basis of neuron morphology. Under standard well-fed laboratory conditions, *C*. *elegans* develops from an embryo to a reproductive adult within three days [9]. The FLP and PVD neurons reach their maximum outgrowth during the fourth larval stage (L4), leading to a tiled series of dendrite branches along the anterior and posterior body wall, respectively (Fig 1A) [7,10,11].

Under conditions of reduced food availability and high population density, *C*. *elegans* can enter into an alternative juvenile stage called dauer [12]. The developmental decision to become dauer relies on parallel insulin signaling and TGF-β pathways, which then influence steroid hormone signaling [13,14]. The insulin signaling pathway integrates nutrient availability and regulates larval development [15]. The insulin receptor, DAF-2 responds to food availability and acts to inhibit the FOXO transcription factor, DAF-16 [16–18]. In opposition to DAF-2, PTEN/DAF-18 acts to positively regulate dauer development by promoting DAF-16 import into the nucleus [19]. Once activated, DAF-16 triggers the synthesis of sterol ligands via the cytochrome p450 homolog DAF-9 to promote reproductive development [20–22]. This signaling cascade acts as a tipping point between reproductive and dauer development and results in remodeling of nearly every tissue in the nematode.

We previously demonstrated that the inner-labial (IL2) neurons undergo dendrite arborization during dauer formation [3]. During non-dauer stages, the IL2s are simple bipolar neurons with a single unbranched dendrite [23]. During dauer formation, the four subdorsal and subventral IL2s undergo extensive branching (Fig 1A and 1B). When returned to favorable environmental conditions, dauers recover to resume the standard developmental course towards adulthood. During dauer recovery, the IL2 arbors are resorbed and return to their non-dauer morphology.

We previously demonstrated that the proprotein convertase KPC-1 is required for both dauer-specific IL2 and PVD/FLP arborization [3]. Subsequent work on the PVDs demonstrated an interaction between KPC-1 and DMA-1, which encodes a membrane-bound leucine-rich repeat receptor [24,25]. DMA-1 acts as part of a protein complex with the epidermally-localized L1CAM homolog SAX-7 and MNR-1, a conserved transmembrane protein [26,27]. Concurrently, the diffusible LECT-2, homologous to leukocyte cell-derived chemotaxin 2, is secreted from surrounding muscle to increase the binding affinity of the DMA-1 complex [28]. The DMA-1 complex in the PVDs and FLPs is regulated through diverse cell functions such as the unfolded protein response and membrane trafficking; disruption of these pathways results in dendrite morphology defects and mislocalization of DMA-1::gfp [29–32]. Furthermore, an interaction between the unfolded protein response and insulin signaling was demonstrated to regulate PVD arbors [30,33].

Here, we use both forward and reverse genetics to demonstrate that the IL2s use shared and distinct molecular mechanisms from the PVDs and FLPs to control arborization. Specifically, we find that while the PVDs, FLPs, and IL2s all use the DMA-1 complex, the regulation of this complex differs between the PVD/FLPs and IL2s. Given the shared location of the FLPs and IL2s in the head of *C*. *elegans*, we examined the structure of the FLPs throughout development and observed architectural differences between the anterior FLPs and IL2s and the posterior PVDs, emphasizing the importance of neighborhood in determining neuronal structure. Finally, we uncover temperature-mediated regulation of the unfolded protein response (UPR) sensor IRE-1 in IL2 branching and describe a battery of new and previously described dauer-remodeling genes that control arborization in the IL2s.

## Results

### The DMA-1 complex is required for dauer-specific IL2 arborization

To determine mechanisms controlling dauer-specific IL2 arborization, we performed a mutagenesis screen. From this screen, we identified a recessive mutation *cbc1*, which leads to disorganized and truncated IL2 branching during dauer with complete penetrance (Fig 1C and 1D). Using traditional genetic mapping, we placed *cbc1* near *dma-1*, a leucine-rich receptor encoding protein required for branching in the PVD neurons [34]. We found that *cbc1* fails to complement the deletion mutant *dma-1(tm5159)* for IL2 branching (11/11 *cbc-1*/*tm5159* dauers defective, S1A Fig). The severity of branching defects in *dma-1(cbc1)* animals suggests that *dma-1(cbc1)* is a strong reduction-of-function allele. Subsequent sequencing of the *dma-1(cbc1)* locus identified a single T to A missense mutation resulting in a leucine to histidine change within the eighth leucine-rich repeat of DMA-1 (Fig 1E).

DMA-1 acts cell-autonomously in the PVDs to regulate arborization and localizes to the PVD dendrite [34]. Similarly, IL2-specific expression of DMA-1 partially rescued the dendrite phenotype in *dma-1(cbc1)* dauers (Fig 1F and S1B Fig). Furthermore, we observed prominent *dma-1p*::*dma-1*::*gfp* expression in the IL2 dendrites during dauer (Fig 1G). Expression of *dma-1p*::*dma-1*::*gfp* was not detectable in adult IL2s (n = 20). Altogether, our results suggest that DMA-1 is acting similarly to control both PVD/FLP and dauer-specific IL2 arborization.

**Fig 2:**
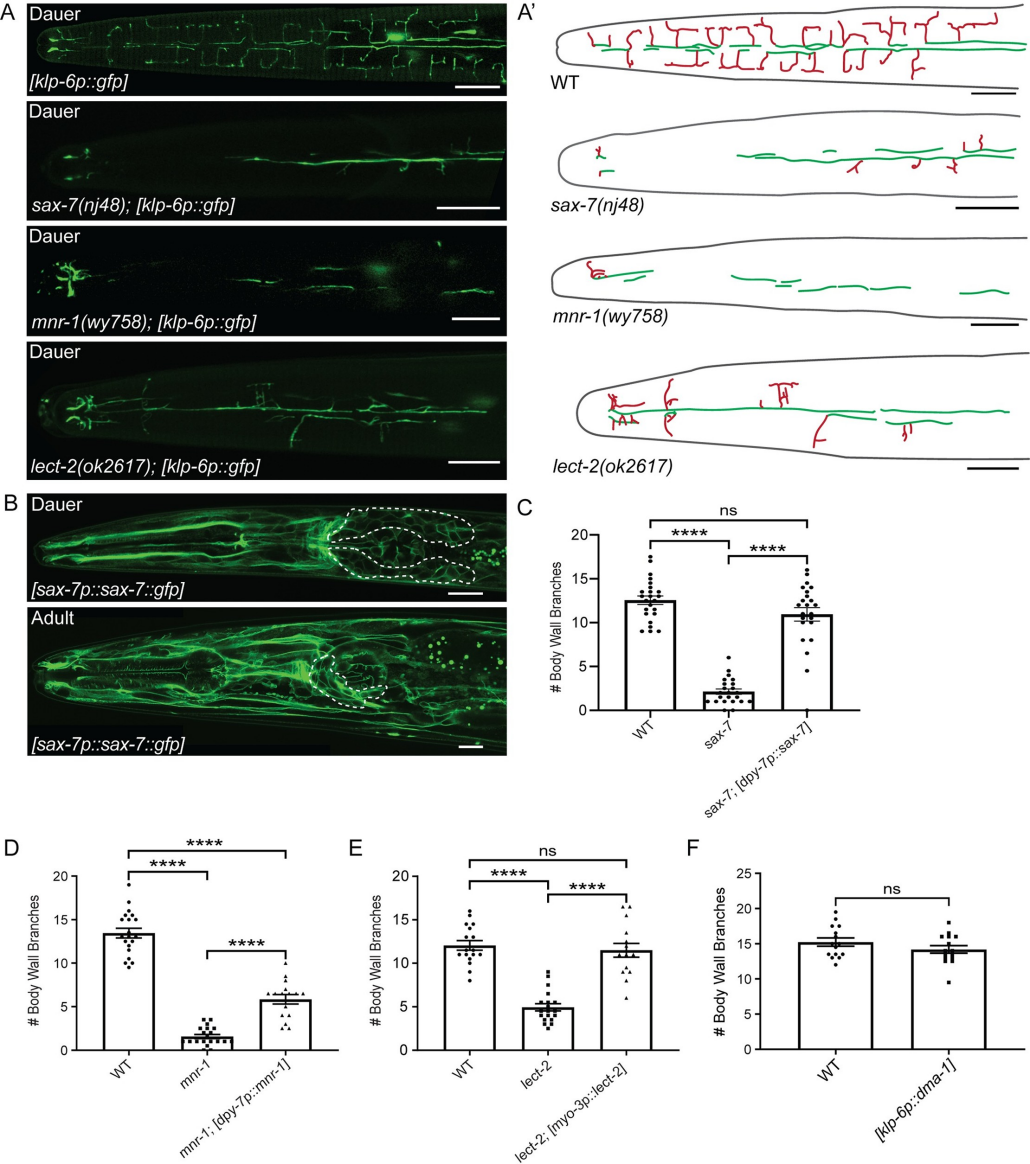
The DMA-1 binding partners are required for dauer-specific IL2 remodeling. (A) Crimeonfocal micrograph of dauers expressing gfp in the IL2 neurons. Images are z-projections focused on the branches at the body wall. Wild-type dauers have extensive IL2 arbors. sax-7(nj48), mnr-1(wy758), and lect-2(ok2617) mutants all have reduced IL2 arborization. IL2 neurons are labeled with *klp-6p::gfp*. (A’) Schematic drawings traced from accompanying micrographs, dorsal midline (green), and body wall branches (red). (B) Confocal micrographs of *sax-7::gfp* expression under its endogenous promoter during dauer (top) and adult (bottom). A dorsal-ventral view of a dauer from a single z-plane shows widespread expression of *sax-7p::sax-7::gfp* in the cell membranes of neuronal (anterior ganglia indicated by dashed line) and non-neuronal tissues. A lateral view of an adult shows a similar expression pattern to the dauer. (C) Quantification of the number of IL2 body wall branches during dauer in *sax-7* mutants and epidermal-specific (*dpy-7p*) rescue of *sax-7*. WT (n = 23), *sax-7* (n = 19), and *sax-7; dpy-7p::sax-7* (n = 19). (D) Expression of *mnr-1* in the epidermis using the *dpy-7* promoter rescues the *mnr-1* mutant branching phenotype. Quantification of the number of IL2 branches along the body wall during dauer in wild-type (n = 21), *mnr-1*(n = 20), and *mnr-1; dpy-7p::mnr-1* (n = 16). (E) Expression of *lect-2* in the body wall muscle using the *myo-3* promoter rescues the *lect-2* mutant branching phenotype. Quantification of the number of IL2 branches along the body wall during dauer in wild-type (n = 19), *lect-2* (n = 19), and *lect-2; myo-3p::lect-2* (n = 16). (F) Quantification of the number of IL2 branches along the body wall during dauer in WT (n = 15) and IL2-specific overexpression of DMA-1 (n = 17). For rescue experiments (C-E), an ANOVA followed by Tukey’s test for multiple comparisons was used to determine significance. For DMA-1 overexpression (F), a t-test was used to determine significance. ****p ≤0.0001 and “ns” p >0.05. Error bars, standard error of the mean. Scale bars, 10 μm.

In the PVDs, DMA-1 acts through a protein complex to control branching [27,28,35,36]. *sax-7* encodes a cell adhesion molecule, homologous to mammalian L1CAM, which acts in the epidermis surrounding the PVDs to control arborization [26,27]. Loss of *sax-7* leads to reduced and disorganized PVD arborization [26,27]. Similarly, we found that *sax-7* is required for dauer-specific IL2 arborization (Fig 2A). Examination of a *sax-7p*::*sax-7*::*gfp* reporter revealed widespread expression in dauers and non-dauers, including neuronal and non-neuronal tissues (Fig 2B) [37]. Similar to the PVDs [26,27], we found that epidermal-specific expression rescued the IL2 branching phenotype in *sax-7* dauers (Fig 2C and S1C Fig). The conserved transmembrane protein MNR-1 functions with SAX-7 in the epidermis to control branching [28,36]. We found that *mnr-1* mutants are also defective for dauer-specific IL2 branching and that these defects could be partially rescued through epidermal-specific expression (Fig 2A, 2D, and S1D Fig). Finally, the muscle-secreted protein LECT-2 interacts with the DMA-1 complex to promote PVD branching [28,36]. Consistent with this, we found that loss of *lect-2* causes defects in dauer-specific IL2 branching that can be rescued through muscle-specific expression (Fig 2A and 2E). Together, our data suggest that the DMA-1 complex is a common tool for branching in all highly arborized neurons of *C*. *elegans*.

Due to the central role of DMA-1 in IL2 arborization, we were interested if DMA-1 expression alone was sufficient to generate branching. Previous work showed that overexpression of DMA-1 in the PVDs leads to supernumerary branching [34]. However, we found that expression of an IL2-specific DMA-1 construct in a wild-type background did not produce extra branches during dauer (Fig 2F). Similarly, we found that overexpression of DMA-1 in the IL2s during adult did not induce IL2 branching in non-dauers (n = 0/17 animals examined) (S1E Fig).

### The PVDs, FLPs, and IL2s have distinct branching architectures

The neighborhood where a neuron is located can lead to differences in branching [38]. As the FLP dendrites share a similar receptive field as the IL2s, we wanted to examine the coordination of branching between these two cell types. The lack of an FLP-specific reporter and the obvious stereotypical morphology of the PVDs has meant that the FLPs have received less attention than PVD arborization. We therefore used Airyscan super-resolution microscopy to examine the architecture of FLP neurons throughout development. We found that the adult FLPs, adult PVDs, and dauer IL2s have distinct dendritic architectures compared to each other.

**Fig 3:**
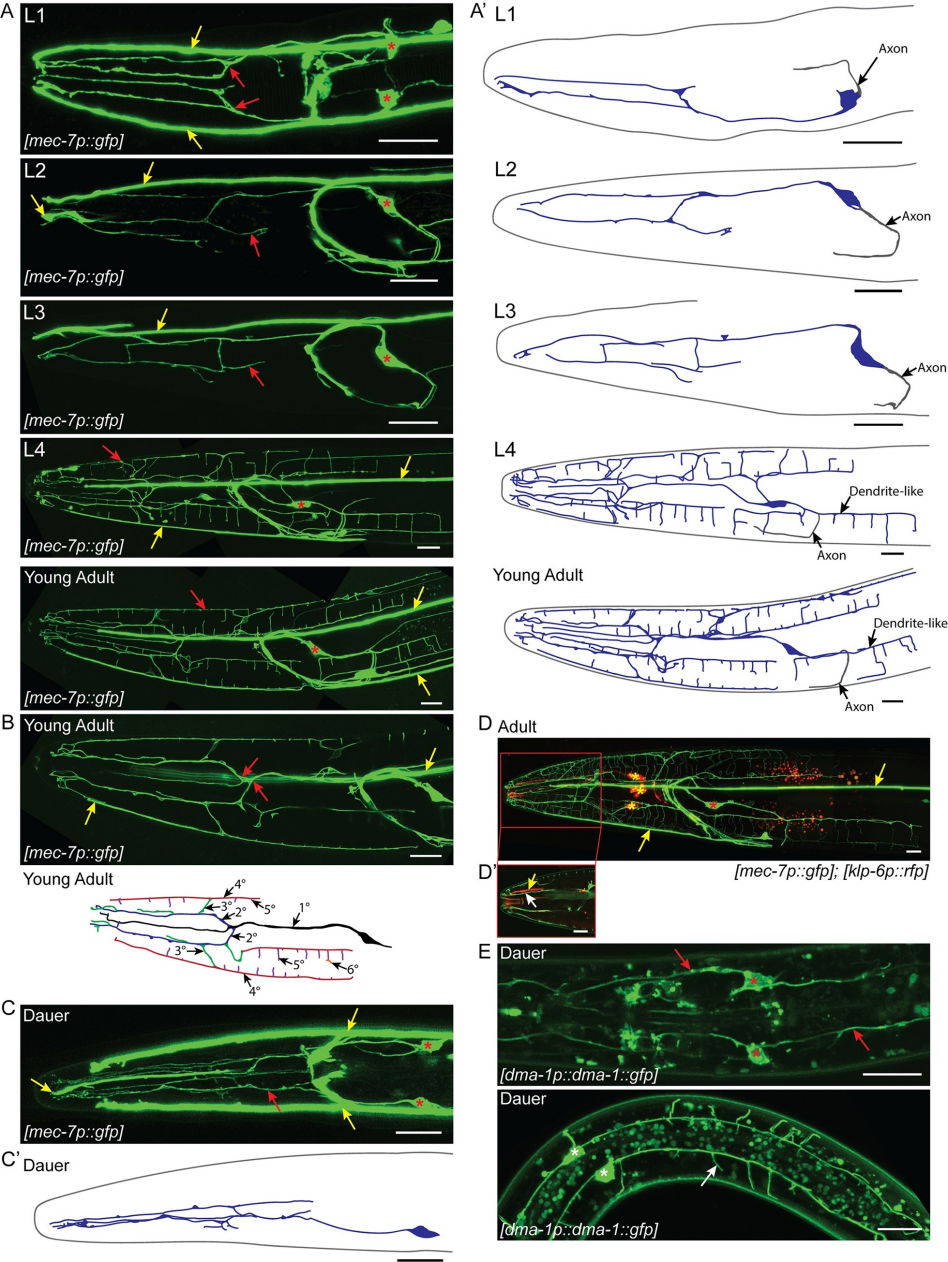
FLP branching architecture. (A) Z-projection confocal micrographs of FLP neurons throughout larval development. Dorsal-ventral view of the FLP dendrite in L1. Each FLP primary dendrite ranges from the cell body to anterior of the metacorpus where it branches to send processes towards the nose. Lateral views of the L2, L3, and L4 FLP dendrite with the focal plane centered around the left FLP arbor. Little additional branching occurs during L2 and L3. The FLPs arborize rapidly during L4. Lateral view of the adult FLP arbors showing extensive dendritic arborization covering the head of the nematode. The primary dendrite travels along the lateral sensory neuron fascicle until anterior of the metacorpus. There it branches and sends secondary processes to the subdorsal and subventral sensory neuron fascicles. A thin primary dendrite continues within the lateral sensory fascicle towards the nose. The secondary dendrites in the subventral and subdorsal fascicles branch again to send processes to the dorsal or ventral midlines and sublateral lines along the body wall where they branch to form the body wall candles of the arbor. Red arrows indicate part of the FLP dendrite. Red asterisks indicate FLP cell bodies and yellow arrows indicate the longitudinal process of the ALM/AVM neurons. (A’) Schematic cartoons traced from the accompanying micrographs for each developmental stage. The left FLP cell bodies and dendrites are shown in blue. The right FLP and ALM/AVM neurons are excluded for clarity. Axonal processes are shown in gray. (B) Z-projection confocal micrograph (top) from a subset of slices of a young adult showing branching of the FLP primary dendrite. Red arrows indicate points where the primary dendrite divides to form secondary dendrites within the subdorsal and subventral sensory neuron fascicles. Yellow arrows indicate ALM/AVM neurites. Illustration of the FLP arbor (bottom) demonstrating the hierarchy of branching. Based on the young adult micrograph, some details have been excluded for clarity. The primary dendrite (black) extends to the nose of the animal following the lateral fascicle. Secondary dendrites (blue) branch from the primary dendrite towards the subdorsal and subventral fascicles, extending parallel to the primary dendrite. Tertiary branches (green) extend radially from the secondary branches towards the dorsal, ventral, and lateral midlines. Quaternary branches (red) extend anterior-posterior along the dorsal, ventral, and lateral midlines. Fifth order branches (purple) extend out over the muscle quadrants from each midline. Occasionally, there is additional branching across the surface of the muscle, a sixth order branch is shown in orange. (C) Dorsal-ventral view of the dauer FLP arbors showing minimal branching compared to the adult. Dauers have no FLP body wall branches. Asterisks indicate the FLP cell bodies, red arrows indicate the FLP dendrite, and yellow arrows indicate the ALM/AVM neuronal processes. (C’) Schematic cartoon of the left FLP (blue) during dauer. (D) Co-labeling of IL2 neurons (red) and the FLP neurons (green). Yellow asterisks indicate IL2 cell bodies while red asterisks indicate the FLP cell bodies. Yellow arrows indicate ALM/AVM neuronal processes. (D’) A single image slice showing the proximal relationship between the FLP dendrite (white arrow) and IL2 dendrite (yellow arrow) in the subdorsal sensory neuron fascicle. The FLPs are labeled with *mec-7p*::*gfp*, which also labels the ALM and AVM touch receptor neurons. The IL2s are labeled with *klp-6p*::*tdTomato*. (E) Z-projection confocal micrograph of *dma-1p*::*dma-1*::*gfp* expression during dauer. These image substacks include the area surrounding the FLP (top) and PVD (bottom) cell bodies respectively. During dauer, *dma-1p*::*dma-1*::*gfp* localizes to the FLP cell body (red asterisks) and dendrite (red arrows) in the head and the PVD cell body (white asterisks) and dendrites (white arrow) in the midbody. Scale bars, 10 μm.

The FLPs, which are born embryonically, have already established their primary dendrite prior to hatching [23]. However, we find that unlike the L2 PVD and non-dauer IL2 unbranched primary dendrites, the FLP primary dendrite has a single branch (1°) anterior of the metacorpus during early development (L1-L3). The early FLP morphology, forms two parallel, longitudinal processes extending to the nose that likely travel in the subdorsal and subventral sensory neuron fascicles (Fig 3A and 3B). From L1 to L3, a single posterior process extends from the cell body and curves to connect into the axon-dense nerve ring; we consider this to be the axon. Similar to previous descriptions, we found little additional arborization of the FLPs until L4 when rapid growth of both the FLPs and PVDs commence [7,10,11]. When fully arborized, the FLP arbor covers the head of the nematode. The primary dendrite (1°) extends a single process from the anterior end of the cell body. The primary dendrite travels with the lateral sensory fascicle until anterior of the metacorpus where it branches twice (2°) to extend three parallel dendrites in each of the subdorsal, lateral, and subventral sensory fascicles. Each subdorsal and subventral dendrite branches several times to send 3° branches out to the dorsal and ventral midlines, respectively. Additionally, both subdorsal and subventral dendrites send branches (3°) to the lateral midline. The dorsal, ventral, and lateral midline branches (4°) extend along the body wall where they branch again, forming body wall branches (5°) over the muscle quadrants. The body wall branches extending from the lateral and dorsal/ventral midline are similar to the “candles” of the PVD menorah [11]. However, unlike the PVD candles, the FLP branches extend towards each other from the both the dorsal/ventral and lateral midlines within each body wall quadrant. As a result of several branch points before the FLP dendrites reach the body wall, the FLPs have five to six orders of branching (Fig 3B). Interestingly, we observed that the neurite, and presumptive axon, originating from the posterior end of the FLP cell body also branches, forming a branched candle structure in the subventral quadrant (Fig 3A). In contrast, while the IL2 axon extends additional processes during dauer, they are not extensive and the PVD axons do not branch [3,7,10,11].

As the FLPs share a receptive field with the IL2s, we were particularly interested in the structure of the FLPs during dauer. Although several neurons have dauer-specific morphology [3,39,40], we found that the FLPs appear to retain their L2 morphology (Fig 3C). A similar finding was recently described in the PVDs during dauer [41]. While the non-dauer IL2 dendrites fasciculate with the lower order FLP longitudinal dendrites, the body wall branches of IL2 dauers and FLP non-dauers are strictly separated by development stage (Fig 3D). Finally, we wished to test whether starvation per se would have subsequent effects on dendrite branching. Animals hatched in the absence of food arrest development as L1s. We tested whether this would have subsequent effects on dendrite arborization. However, we saw no obvious differences to adult FLP or dauer IL2 branching following recovery from L1 arrest (S2 Fig). Our analysis of FLP and IL2 branching suggests both neighborhood effects, which create similarities in branch patterns, as well as developmental coordination between the two neuron classes.

### Regulatory mechanisms controlling DMA-1 localization differ between IL2 and PVD/FLP arbors

We hypothesized that *dma-1* would be downregulated in the FLPs during dauer to prevent inappropriate arborization. To our surprise, we found that the *dma-1p*::*dma-1*::*gfp* translational reporter is prominently expressed in the FLPs and PVDs during dauer (Fig 3E). This result suggests there are additional factors inhibiting FLP and PVD branching in dauers. We, therefore, examined known regulators of the DMA-1 complex. Exocytosis of DMA-1 from the endoplasmic reticulum to the PVD dendritic membrane uses the RAB-like GTPase, RAB-10 and an ortholog to human exocyst complex component 8, EXOC-8. Loss of *rab-10* and *exoc-8* lead to reduced PVD arborization [31,32]. Interestingly, we found that *rab-10* and *exoc-8* are dispensable for dauer-specific IL2 arborization (S3A Fig). Furthermore, we found that *dma-1*::*gfp* localizes to the IL2 dendrite in the *rab-10* mutant, similar to its localization in the wild type background (S3D Fig). We hypothesized that a RAB-10 paralog could function in the IL2s to transport DMA-1 to the membrane during dauer. Using a phylogenetic examination of human Rab GTPases as a guide [42], we examined mutants of the *rab-10* paralogs *rab-8* and *rab-35* for effects on IL2 arborization. We found no effect from the loss of *rab-8* or *rab-35* on IL2 arborization (S3B Fig).

**Fig 4:**
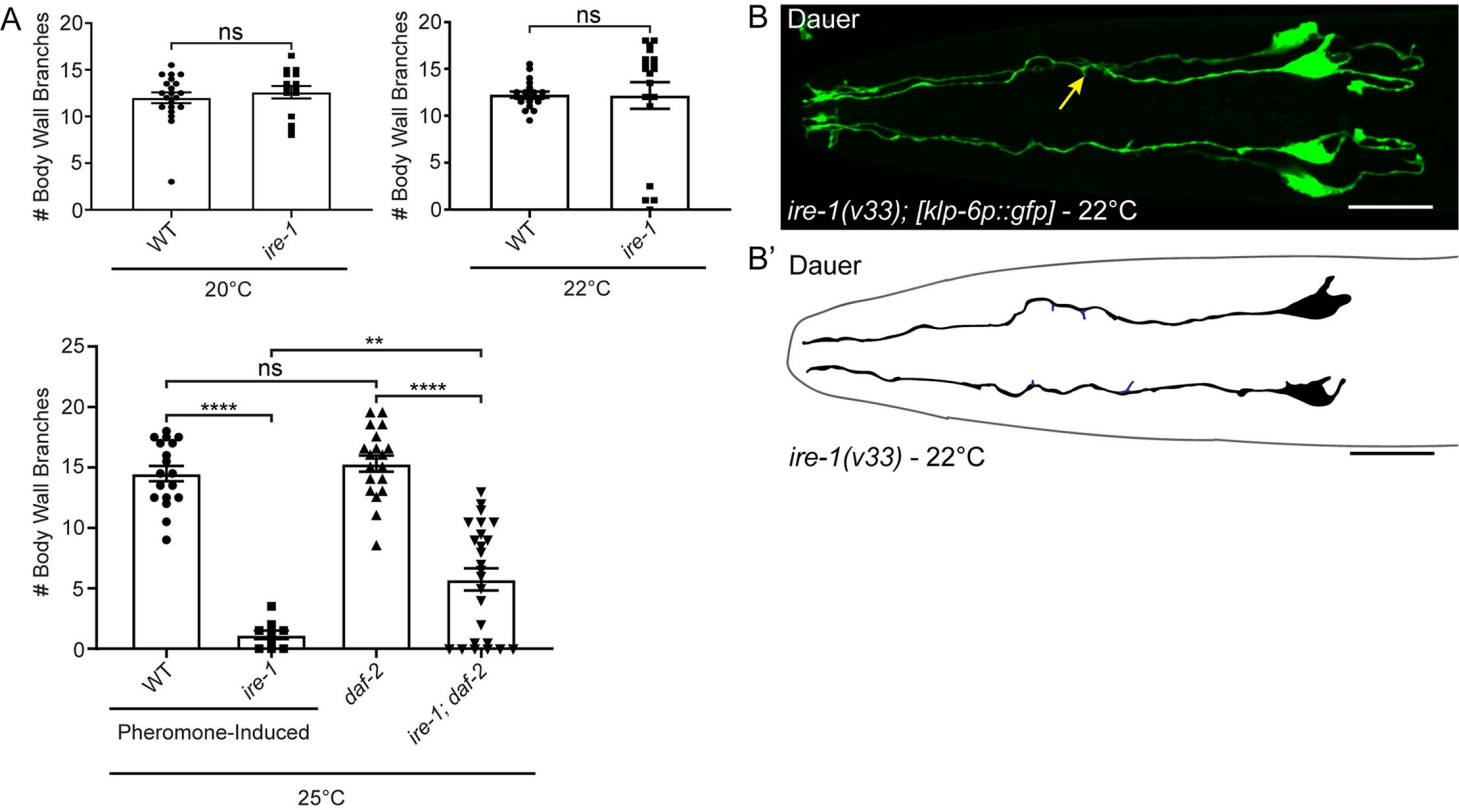
IRE-1 is required for IL2 remodeling during dauer formation. (A) Quantification of *ire-1* IL2 arbors at multiple temperatures. At 20°C (n = 15) and at 22°C (n = 19), *ire-1* mutants show no statistical difference in the number of body wall branches compared to wild-type using a parametric t-test (n = 20 and n = 21, respectively). However, at 22°C, 21% of the *ire-1* mutants show severe branching defects with fewer than three body wall branches. At 25°C, *ire-1* mutant dauers (n = 9) show a significant decrease in branch number compared to the wild-type dauer control (n = 18). The *ire-1; daf-2* double mutant (n = 25) have significantly fewer body wall branches than the *daf-2* control (n = 19). Student’s t-test was used to calculate p-values at 20° and 22°C. ANOVA followed by Tukey’s test for multiple comparisons were used to compare branching at 25°C. **p ≤0.01, ****p ≤0.0001, and “ns” p >0.05. Error bars, standard error of the mean. (B) Z-projection micrograph dorsal-ventral view of an *ire-1* mutant dauer with truncated radial branches (yellow arrow) and lacking all midline and body wall branches. IL2 neurons are visualized by *klp-6p::gfp*. (B’) Schematic illustration of the *ire-1(v33)* mutant. Primary dendrite and cell body are shown in black and truncated radial dendrites in blue. Scale bar, 10 μm.

Similar to the requirement for RAB-10, the unfolded protein response (UPR) sensor IRE-1 is needed for proper localization of DMA-1 and arborization in the PVD neurons [29,30]. Loss of IRE-1 leads to high penetrance defects in PVD branching, but it was suggested that *ire-1* mutants were normal for IL2 branching [29]. While the average number of IL2 branches in *ire-1* mutant dauers did not differ statistically from wild-type at 22°C (Fig 4A) using the t-test, we noticed that 21% of *ire-1* dauers (n = 4/19) lacked almost all IL2 dendritic branching (Fig 4B). When analyzed as a binary statistic comparing the fraction of animals having defective branching, we found a statistically significant difference between wild-type and *ire-1* mutant dauers at 22°C (Fisher’s Exact Test, p = 0.0471).

During the course of our studies, we observed that populations of *ire-1* mutants crashed if not stored in a temperature-controlled incubator and that there was an apparent correlation between culture viability and fluctuations in room temperature. We hypothesized that dauer-specific branching in *ire-1* mutants is temperature-sensitive. Consistent with this, we found no defects in IL2 arborization at 20°C (Fig 4A). We were unable to observe *ire-*1 mutant dauers grown under starvation conditions at 25°C; at this temperature, *ire-1* mutants were sterile with variable developmental defects. Therefore, to determine if higher temperatures would increase branching defects, we reared *ire-1* mutants on dauer pheromone [43]. Following exposure to high concentrations of crude dauer pheromone at 25°C, 6% (n = 46) of *ire-1* mutants underwent dauer development. The remainder arrested at earlier developmental stages. In contrast, 91% (n = 46) of wild-type embryos grown at 25°C developed into dauer on pheromone plates within three days. Consistent with a temperature-sensitive response, we found severe IL2 defects in 100% (n = 9) of *ire-1* dauers grown at 25°C (Fig 4A). These results suggest that *ire-1* is not needed under optimal temperature conditions, but is indispensable for IL2 branching under elevated temperatures.

Previous research showed that the transcription factor XBP-1, which is activated through excision of an intron by IRE-1 [44], is not necessary for dendritic branching in the PVDs [27]. Similarly, we found that *xbp-1* is not needed for IL2 branching during dauer (S3C Fig). We were curious if other factors contributed to the UPR regulation of dauer. To determine if the UPR was activated differently during dauer, we examined mutants of other UPR-related genes including *skr-5*, *tps-1*, *gst-4*, and *F47F2*.*1* [33,45,46]. However, all other UPR candidates examined were able to form complete IL2 arbors during dauer (S3C Fig).

In the PVDs, loss of *ire-1* leads to mislocalization of *dma-1*::*gfp* to the endoplasmic reticulum (ER) [29]. To determine if the IL2 defects in *ire-1* mutants are a result of an abundance of DMA-1 in the ER, we looked at the localization of *dma-1p*::*dma-1*::*gfp* in the *ire-1* mutant grown at 22°C. We found no obvious difference in *dma-1*::*gfp* localization between *ire-1* mutant dauers and wild-type (S3D Fig). Therefore, it is possible that *ire-1* is acting through a DMA-1-independent mechanism to enable the IL2s to arborize at high temperatures.

The PVD branching defects in *ire-1* mutants are completely rescued by disruption of the DAF-2 insulin signaling receptor [30]. Dauer formation acts through the insulin signaling pathway and loss of *daf-2* leads to constitutive dauer formation [17]. We examined *ire-1* dauers in a *daf-2* temperature-sensitive genetic background. Similar to *ire-1* single mutants, at 25°C *ire-1; daf-2* mutants have a significant decrease in the number of body wall branches compared with *daf-2* mutants alone (Fig 4A). Although branching in the *ire-1; daf-2* double mutant was improved compared with the *ire-1* mutant alone at 25°C, these data suggest that unlike in the PVDs, reduced insulin/IGF1 signaling does not completely bypass the requirement for *ire-1* in the IL2s at elevated temperature.

As *daf-2* partially rescued the *ire-1* IL2 remodeling defects, we explored the insulin-signaling pathway in greater depth. If *daf-2(e1370)* mutant adults are transferred to 25°C, they become reproductively quiescence and exhibit some dauer-like morphology and behaviors [47]. We hypothesized that loss of *daf-2* in post-dauer animals would be sufficient to induce IL2 branching. We transferred L4 *daf-2* animals to 25°C and checked for ectopic IL2 growth for three consecutive days. Although we reproduced the reproductive quiescence phenotype, the IL2 dendrites remained unbranched (n = 0/60 animals examined). Similar to our finding that expression of DMA-1 outside of dauer does not induce IL2 branching, these results suggest a potent inhibition of IL2 branching outside of the dauer stage.

### DAF-16 is necessary for IL2 arbor formation during dauer, but not FLP arbor formation in adult animals

**Fig 5:**
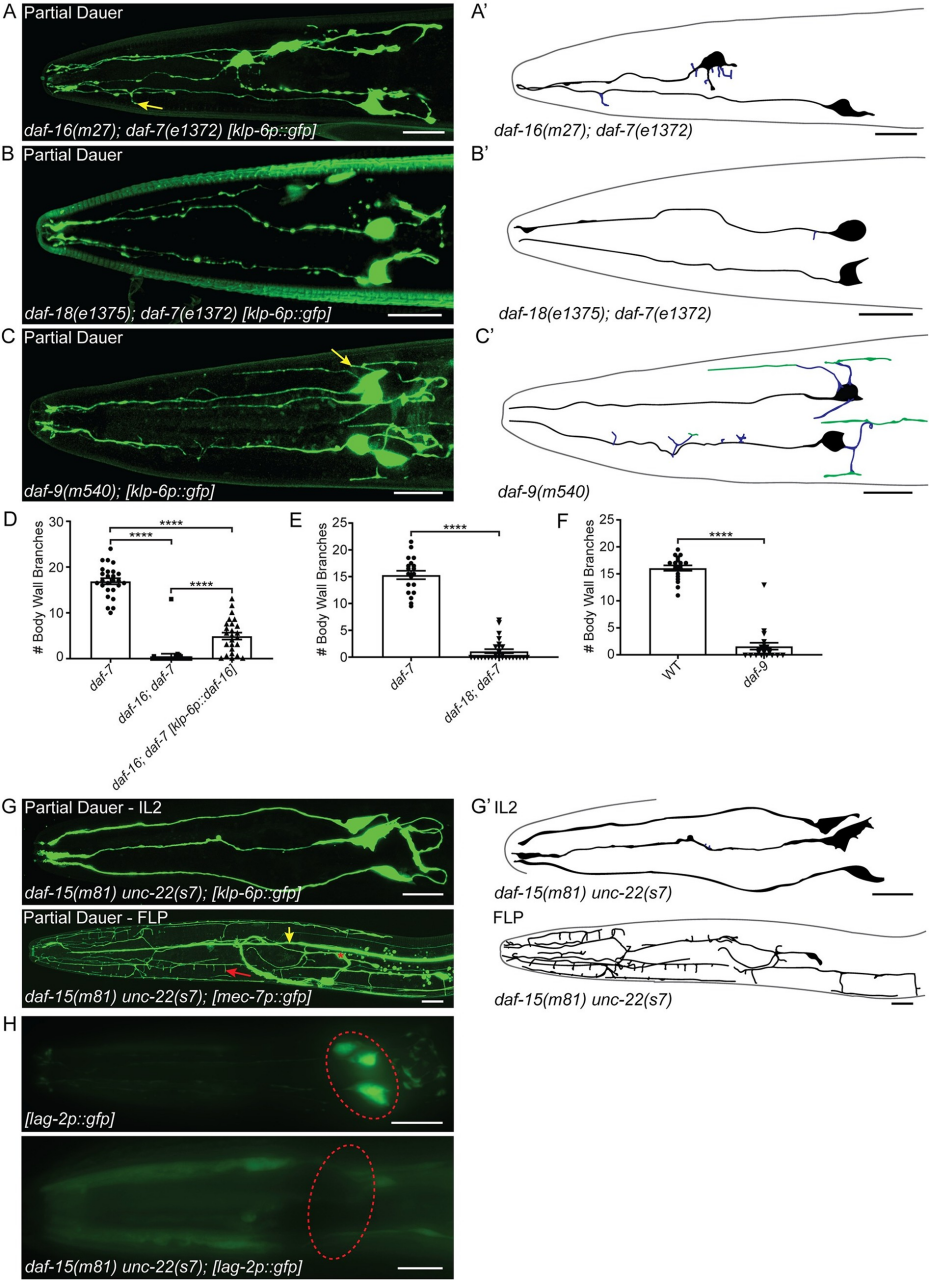
Genes regulating dauer formation are required for IL2 and FLP arborization. (A) Z-projection confocal micrograph of *daf-16(m27); daf-7(e1372)* partial dauer showing few truncated secondary IL2 branches (yellow arrow). (A’) Schematic of *daf-16(m27); daf-7(e1372)* IL2 arbors. IL2 cell bodies and primary dendrites (black) show displacement defects within the head of the nematode. There are few secondary branches (blue). (B) Z-projection confocal micrograph of a *daf-18(e1375); daf-7(e1372)* partial dauer with a lack of IL2 body wall branches. (B’) Schematic of *daf-18(e1375); daf-7(e1372)* IL2 arbors. IL2 cell bodies and primary dendrites (black) and a single truncated radial branch (blue). (C) Z-projection confocal micrograph of *daf-9(m540)* partial dauer. *daf-9* partial dauers extend several branches directly from the cell body (yellow arrows), but form few higher-order branches. (C’) Schematic of *daf-9(m540)* IL2 arbors. Radial branches (blue) extend from both the primary dendrite and the cell body (black), some midline branches (green) form proximal to the cell body. (D-F) Quantification of IL2 body wall branches. (D) The *daf-16(m27); daf-7(e1372)* mutant (n = 27) is severely defective in IL2 arbor formation compared to the *daf-7* control (n = 26). Expression of *daf-16* in the IL2 neurons with a *klp-6* promotor (n = 25) is sufficient to increase arborization. (E) *daf-18(e1375); daf-7(e1372)* partial dauers (n = 29) have fewer body wall branches compared to the *daf-7* control (n = 19). (F) *daf-9(m540)* mutants (n = 21) have fewer body wall branches compared to the wild-type control (n = 19). (G) Z-projection confocal micrograph of *daf-15(m81) unc-22(s7)* partial dauer showing completely unbranched IL2 neurons (top) and extensive branching of the FLP neuron (bottom), including body wall branches (red arrow). Red asterisk indicates FLP cell body, yellow arrow indicates ALM neurite. (G’) Schematics of the *daf-15* IL2 and FLP arbors. The FLP dendrite branches to cover the anterior of the head with fewer branches near the cell body. (H) Epifluorescent z-projections of the anterior of a wild-type (top) dauer and *daf-15(m81) unc-22(s7)* (bottom) partial dauer with *lag-2p::gfp* that labels the IL2s during the dauer stage and no other stages [53]. We use the *lag-2p::gfp* reporter as an indicator of the developmental identity of the IL2 neurons. Red dashed-line circle indicates the relative position of the IL2 cell bodies. For analyses between two groups, statistical significance was determined with t-tests (E and F). For genetic rescue comparisons, statistical significance was determined with ANOVA followed by Tukey’s test for multiple comparisons (D). ****p ≤0.0001. Error bars, standard error of the mean. IL2 neurons are visualized by *klp-6p::gfp* (A, B, C, and G) and FLP neurons are visualized by *mec-7p::gfp* (G). Scale bars,10 μm.

The compensation of the *ire-1* mutant branching phenotype in the PVDs through reduced insulin/IGF signaling is dependent upon the FOXO transcription factor DAF-16 [30]. Disruption of DAF-16 causes defects in dauer formation; however, *daf-16* mutants will form partial dauers in a *daf-*7 dauer-constitutive (*daf-*c) mutant background [48,49]. *daf-16* partial dauers display some, but not all of the characteristic tissue remodeling found in wild-type dauers [48]. We found that *daf-16; daf-7* partial dauer IL2 arbors were severely reduced compared to *daf-7* dauers (Fig 5A and 5D). We also found frequent defects in cell body positioning and abnormal axon morphology in *daf-16; daf-7* partial dauers. However, we found no apparent defects in FLP arbor formation in *daf-16; daf-7* adult animals (S4A Fig) suggesting that DAF-16/FOXO differentially regulates arborization in the IL2s and FLPs.

DAF-16 is widely expressed in multiple tissue types [16]. Previous work showed that pan-neuronal rescue of DAF-16 is sufficient to rescue all dauer remodeling and formation defects in the *daf-16* mutant [50]. To determine if *daf-16* is functioning cell-autonomously, we drove expression in the IL2 neurons alone. We found a slight, but statistically significant increase in arbor formation following IL2-specific expression of *daf-16* (Fig 5D). While these results suggest a possible cell-autonomous role in *daf-16* regulation of IL2 branching, they do not rule out other mechanisms.

Mutations in several other components of the dauer formation pathway will produce partial dauers either alone or in combination with *daf-c* mutants [51]. The phosphatase and tensin (PTEN) homolog DAF-18 is required for proper dauer formation. Similar to *daf-16*, *daf-18* mutants form partial dauers in a *daf-*c background [48]. We found that *daf-18; daf-7* partial dauers are defective for IL2 arbor formation (Fig 5B and 5E), but wild-type for FLP arborization (S4B Fig). *daf-9* encodes a cytochrome p450 that synthesizes a lipid hormone which binds to a nuclear hormone receptor to bypass the dauer stage. Animals carrying the *daf-9(m540)* mutation constitutively form partial dauers when grown on cholesterol-free media [52]. We found that *daf-9(m540)* partial dauers have a reduced number of branches from their primary dendrite, while still exhibiting a multipolar cell body (Fig 5C and 5F). *daf-9* mutant adults were wild-type for FLP arborization (S4C Fig). Similar to *daf-9*, disruption of the TOR complex associated protein DAF-15 causes arrest of partial dauers. *daf-15* arrested animals have few dauer-like morphologies [53]; however, we found no branching in any *daf-15* partial dauers (Fig 5G). Given the lack of dauer-specific features, we questioned whether these were true dauers. We used a *lag-2p*::*gfp* reporter which labels the IL2s during dauer and no other stages as an additional indicator of dauer identity [54]. Indeed, we found that the *daf-15* mutants fail to display dauer-specific expression of *lag-2p*::*gfp* in the IL2 cell bodies (Fig 5H). This corresponds with a recent publication demonstrating reduction of dauer-specific expression of *ets-10p*::*gfp* in a *daf-15* background [55]. To our surprise, the *daf-15* partial dauer FLPs had extensive arborization (Fig 5G). This was unexpected as the FLPs do not obtain body wall branches until L4. This precocious formation of FLP branching may suggest that TOR complex 1 regulates the timing and dauer-specific inhibition of FLP arborization.

## Discussion

**Fig 6:**
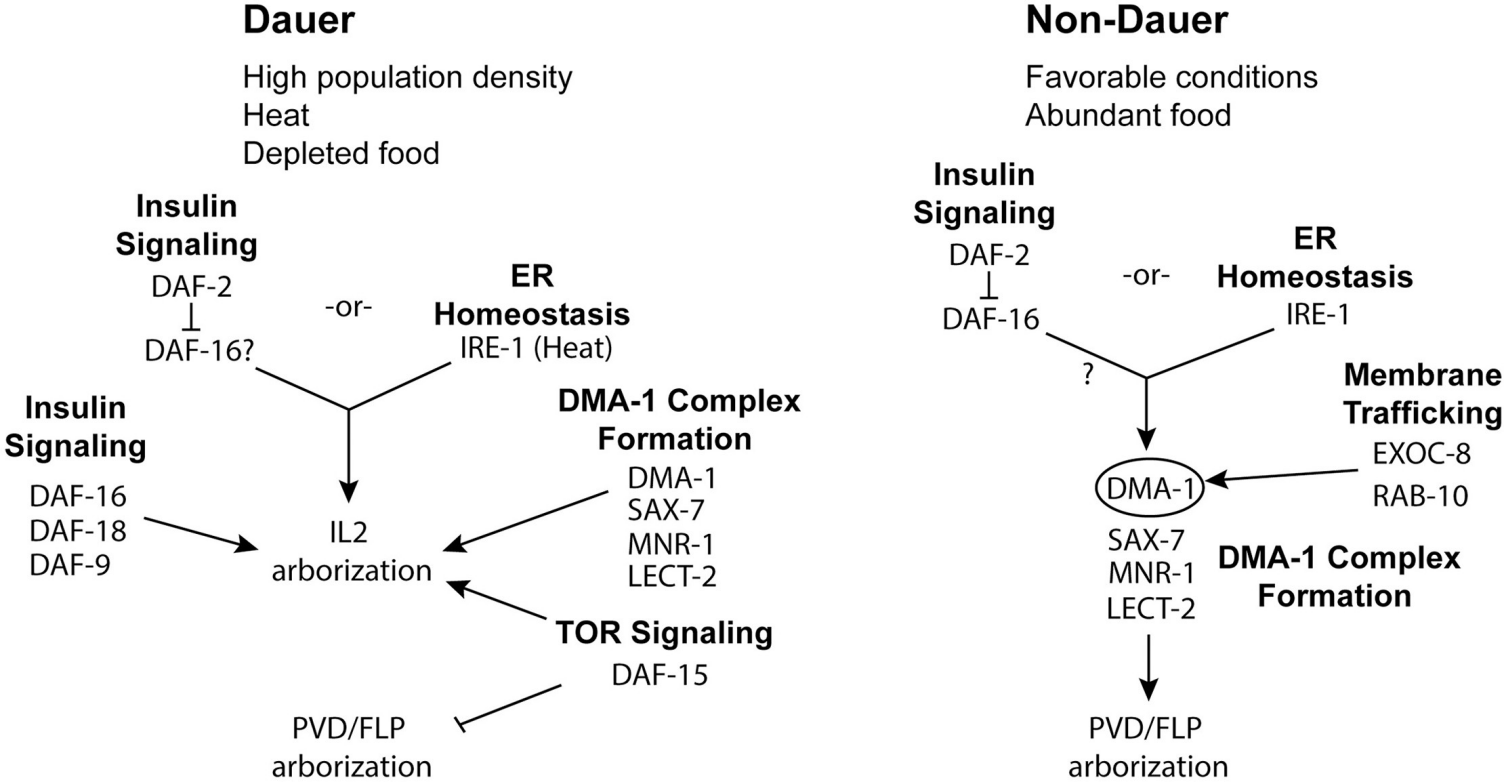
Differential regulation of IL2 branching during dauer compared to PVD/FLP branching in non-dauer animals. Model of the molecular pathways of dendrite arborization during dauer (left) and PVD/FLPs during reproductive development (right). In both cell types, DMA-1 controls arborization. However, in the PVDs, the DMA-1 complex is directly regulated through IRE-1 and membrane trafficking pathways (EXOC-8 and RAB-10) [31]. In contrast, IRE-1 is only needed in the IL2s at elevated temperatures and does not appear to affect DMA-1 localization. Similarly, the EXOC-8 and RAB-10 trafficking pathways are dispensable for IL2 arborization. In non-dauer development, loss of *daf-2* is able to completely bypass the requirement for *ire-1* in the PVDs through activation of DAF-16 [30]. In contrast, loss of *daf-2* only partially rescues the *ire-1* mutant phenotypes in the dauer IL2s. Additionally, DAF-16 is independently required for proper IL2 arborization during dauer. In addition, to DAF-16, mutants in *daf-18* and *daf-9* lead to partial dauer phenotypes that include defective IL2 arborization. Loss of *daf-15* causes a complete lack of IL2 arbors and inappropriate FLP arborization.

We find that the DMA-1 complex is used to regulate branching in each highly arborized neuron in *C*. *elegans* (Fig 6). This highlights the versatility of the DMA-1 receptor-ligand complex. In addition to expression in the FLPs and PVDs, DMA-1 is expressed in the IL2s. However, IL2 expression of DMA-1 is restricted to dauer while the FLP/PVD expression occurs during both dauer and non-dauer stages. Furthermore, we show that the DMA-1 complex is not sufficient to induce branching in the IL2s outside of dauer despite the presence of SAX-7, MNR-1, and LECT-2 during non-dauer stages [26,36]. Similarly, we found that during dauer the FLPs stop branching despite continued expression of DMA-1. Our findings suggest the presence of inhibitors of IL2 branching outside of dauer and FLP branching during dauer and support the hypothesis that activation of DMA-1 is context-dependent [34]. Our finding of extensive FLP branching in *daf-15* partial dauers suggests that the TORC1 complex is required to inhibit FLP branching during dauer. In *Drosophila*, the TORC2 regulates dendritic tiling [56]. In mammals, mTOR localizes to dendrites and is required for local translation [57] and knockdown of mTOR leads to reduced dendrite arborization in cultured hippocampal neurons [58].

Our detailed examination of FLP branching revealed undescribed structural differences between it and the well-studied PVD neurons. Both neuron types extend terminal branches along the body wall between the somatic muscle and surrounding epidermis [7]. Despite the similarity in transcriptional regulation and downstream molecular effectors controlling branching, the lower-order branches differ substantially between PVD and FLP. The PVD secondary dendrites extend regularly from the laterally positioned primary dendrite [7,10]. In contrast the dendritic tree of the FLPs have a less stereotypical branching pattern with irregularly spaced lower order branches (Fig 3B). The presence of FLP body wall branches that extend from both the lateral and dorso-ventral midlines appears intermediate between the PVD and IL2 branching patterns. Neurite formation has long been known to be shaped by surrounding tissues [26–28,36,59,60]. The primary dendrites of the PVDs fasciculate with the relatively sparsely populated lateral nerve containing only 2–3 other neurons [61]. In contrast, the FLP primary dendrite travels within a sensory neuron fascicle with more than 20 neuronal and glial processes. Also, the epidermis surrounding the PVDs comprises a single syncytium, whereas the area of FLP branching comprises multiple epidermal syncytia. These differences in local environments may affect the different architectures seen in each arborizing neuron.

One possible mechanism for the different arborization pattern between the PVD/FLP classes and the IL2s is through the transport of DMA-1. Indeed, we found that, unlike in the PVDs [31], RAB-10 is not required for IL2 arborization. One possible explanation is that the relatively smaller area of the IL2 arbor, compared with the expansive PVD and FLP dendritic trees, does not require a RAB-based membrane transport (Fig 1A). Alternatively, an unknown RAB protein may be required specifically in the IL2s.

Our examination of the *ire-1* mutant suggests that ER-related homeostasis may regulate dauer IL2 remodeling independent of an interaction with the DMA-1 complex. The UPR was previously implicated in dauer formation in a *daf-28 daf-*c mutant background [62]. IRE1 acts as a sensor and activator of the XBP-1 transcription factor [63]. However, similar to findings in the PVDs, we found that *xbp-1* is not required for proper IL2 arborization [29,30]. It is likely that the IL2s also use the XBP-1-independent RIDD (regulated Ire1-dependent decay) pathway. The UPR regulates various aspects of remodeling in mammalian tissues [64,65]. Future work will be needed to determine the role of IRE-1 in dauer remodeling of other tissues, whether it is acting through the canonical UPR or XBP-1 independent mechanisms, and how these systemic stress responses influence dendrite morphology.

Interestingly, we found that the inositol requiring enzyme IRE-1 is only needed for IL2 arborization under elevated temperature (Fig 6). The reason for this conditional requirement for IRE-1 in the IL2s is unclear. One possible explanation is that during dauer additional stress-responsive pathways are upregulated that obviate the need for *ire-1* to regulate DMA-1 transport in non-stress inducing temperatures. In support of this, the effect of *ire-1* mutations in the PVDs is rescued through loss of *daf-2*, which activates signaling of the FOXO transcription factor DAF-16 [33]. DAF-16 activation can bypass the requirement for IRE-1 ER homeostasis in *C*. *elegans* and human cell culture [33]. In dauers, DAF-16 is already activated, thus making IRE-1 redundant until additional stressors, such as elevated temperature, are present. Studies in plants and fungi demonstrated that IRE-1 homologs are conditionally required under elevated temperatures [66,67]. Similarly, double mutants of the UPR-related genes *pek-1* and *xbp-1* in *C*. *elegans* show reduced viability at high temperatures [68].

Dauer formation comprises remodeling of neurons, muscle, and epithelial tissue [69]. In wild-type animals these various tissues must coordinate remodeling to produce a complete dauer. We found that several mutants with known defects in dauer morphology also have defects in IL2 arborization. Unlike the FLPs, DAF-16 is independently required for IL2 arborization in dauers.

*daf-16* partial dauers undergo incomplete remodeling of epithelial and alimentary tissues [48]. DAF-16 is also required for proper axon outgrowth in the *C*. *elegans* AIY interneurons [70]. We add to this by demonstrating a lack of IL2 branching in *daf-16* partial dauers and, similar to its role in AIY outgrowth, we find that DAF-16 is acting cell-autonomously in the IL2s to control dendrite outgrowth. The incomplete rescue of *daf-16* mutants using an IL2-specific promoter may suggest that *daf-16* is controlling arborization through both cell-autonomous and system-wide regulatory pathways.

We also found IL2 branching defects in *daf-9*, *daf-15*, and *daf-18* mutants. Electron microscopy of the anterior ciliated tips of sensory neurons suggested that *daf-9* partial dauer IL2 neurons are intermediate in morphology between dauers and non-dauers [53]. Our data shows that while *daf-9* partial dauer IL2s do branch, they lack almost all terminal body wall branches. *daf-18* encodes the Phosphatase and Tensin (PTEN) homolog and, in combination with *daf-c* mutants, produces partial dauers with an unremodeled pharynx that continues to pump [48]. Because *daf-18* falls upstream to *daf-16* in the dauer formation pathway, we cannot conclusively attribute the defects found in the *daf-18* partial dauer to a lack of DAF-18 alone. Nevertheless, DAF-18/PTEN plays a major role in neuron development. Loss of PTEN in mice leads to hypertrophic arborization in the dentate gyrus [71]. Inhibition of neurite outgrowth by PTEN in mammalian systems acts through mTOR activation [72]. The TOR pathway *daf-15* mutation leads to irreversible dauer formation with very few dauer characteristics [53]. While our analysis questions whether *daf-15* mutants form dauers, additional experiments will be required to understand the roles of these dauer remodeling genes in IL2 arborization.

## Materials and methods

### Maintenance of *C*. *elegans* strains

*C*. *elegans* were cultured and genetic crosses were performed on nematode growth media (NGM) with *E*. *coli* OP50 bacteria as a food source using standard protocols [9]. All strains were maintained at 22°C unless otherwise noted. A complete list of strains used in this study can be found in S1 Table. Dauers were selected from plates that had grown to starvation, one-week after passaging, unless otherwise noted. To generate dauers during a single generation for complementation testing and testing dauer formation in the *ire-1* mutant at 25°C, dauers were grown from eggs on NGM containing crude dauer pheromone and immediately analyzed. Crude dauer pheromone was produced using established techniques [73]. For all experiments performed with a dauer constitutive mutant background, *daf-2* or *daf-*7 animals were grown from eggs in the presence of food at 25°C and immediately analyzed. In all cases, dauers were tentatively identified under the dissecting microscope by their relatively thin body, lack of pharyngeal pumping, and suppression of head foraging movement. The exception to this were partial dauer phenotypes which were taken into account when selecting animals for analysis. Following examination under the compound microscope at 100x magnification with DIC optics, the animals were confirmed to be dauer or partial dauer if they had altered stoma morphology, lateral alae, or a shrunken pharyngeal bulb [69].

### Plasmid constructs and generation of transgenic lines

Some plasmids were generously donated from other labs (S2 Table). Plasmids were built in the Schroeder lab using Gibson Assembly [74]. Rescuing constructs were injected following standard microinjection technique at 5 ng/μl together with coel::RFP at 50 ng/μl and pBluescript to a final concentration of 100 ng/μl [75]. Independent stable lines were isolated for each rescue experiment and analyzed for number of body wall branches. The number of rescuing lines per stable line isolated for each construct are as follows: *[klp-6p*::*dma-1]* (3/3); *[dpy-7p*::*sax-7]* (2/2); *[dpy-7p*::*mnr-1]* (3/3); *[klp-6p*::*daf-16]* (1/1). Successful rescue was determined by a quantifiable change in body wall branch numbers, with ANOVA followed by Tukey’s multiple comparison. For DMA-1 overexpression, we crossed a *[klp-6p*::*dma-1]* rescuing array into the WT background. TV8545 *wyEx3282[dma-1p*::*dma-1*::*gfp*::*SL2*:: *mcherry(fosmid)]; pha-1(e2123 ts)* was used for all [*dma-1*::*gfp*] localization analyses. A complete list of plasmids, and assembly primers can be found in S2 Table.

### Mutagenesis and genetic mapping

*dma-1(cbc1)* was generated using a standard EMS mutagenesis protocol [76] in a *daf-7(e1372) myIs13[klp-6p*::*gfp] daf-c* background. Newly generated mutant strains were backcrossed to wild type at least twice before usage and assigned to a linkage group based on two-factor mapping with EG1020 *bli-6(sc16) IV; dpy-11(e224) V; lon-2(e678) X* and EG1000 *dpy-5(e61)I; rol-6(e187)II; lon-1(e1820)III*. Complementation testing for *dma-1(cbc1)* was performed on dauer pheromone-containing media with *dma-1(tm5159)I*. The *dma-1(cbc1)* lesion was identified with Sanger Sequencing.

### Gene schematics

Gene schematics were constructed using wormweb.org. Functional domains for *dma-1* were determined using LRRfinder.com.

### FLP developmental time course

We used *muIs32[mec-7p*::*gfp]* to visualize FLP cell morphology. To generate synchronous cultures, eggs were collected from gravid hermaphrodites by bleaching with 5% bleach and 5N NaOH in M9 buffer [77]. Eggs were allowed to hatch overnight in M9, generating a population of L1 arrested larva. Arrested L1 larvae were then transferred to NGM plates seeded with OP50 *E*. *coli*. The L1 larvae were allowed to develop at 22°C until the desired stage for imaging. We evaluated the worms after 1, 14, 22, 30, and 40 hours on food to determine FLP morphology at the L1, L2, L3, L4 and young adult stages, respectively. Dauers were selected based on morphology and behavior from recently food-depleted plates. Representative images for each stage were acquired using a Zeiss 880 Airyscan confocal microscope. Twenty animals were observed under an epifluorescence compound microscope at each time point to confirm the consistency of our observations.

### L1 arrest

Gravid hermaphrodites were bleached to isolate eggs using a solution of 5% sodium hypochlorite and 5M NaOH. Eggs were left to hatch in M9 buffer overnight, in the absence of food these L1 animals will arrest development. Following L1 arrest, the arrested animals were allowed to develop to the adult stage on NGM or to develop into dauers on dauer-inducing, pheromone media. Adults and dauers were then examined for dendrite morphology following early life starvation.

### IL2 body wall branch counts

The number of branches originating from either the dorsal or ventral midlines were counted to obtain a body wall branch number. The branch counts were averaged for all cells quantified within an individual. Averages were then compared using either a t-test or ANOVA followed by Tukey’s test for multiple comparisons to determine statistical significance.

### Microscopy

Animals were anesthetized with 100 mM levamisole and mounted on 10% agarose pads containing 20 mM levamisole for sustained immobilization during imaging. Datasets for quantification were gathered with a Zeiss AxioImager microscope equipped with DIC and fluorescence optics. High-resolution representative images were acquired with a Zeiss LSM 880 confocal microscope with Airyscan postprocessing.

### Temperature shift assay

*daf-2(e1370);myIs14[klp-6p*::*gfp]* one-day-old adults were transferred from 15°C to 25°C. Twenty animals were examined for IL2 morphology every 24 hours for three consecutive days.

## Supporting information

S1 Fig The DMA-1 complex is required for IL2 branching during dauer. (A) *dma-1(tm5159)/dma-1(cbc1)* heterozygotes show a reduction of branches. (A’) Schematic drawings traced from the accompanying micrographs indicate the IL2 dendrites that are visible in the images. Dorsal midline branches (green) and body wall branches (red). (B) Quantification of IL2-specific DMA-1 rescue. Replicate #2, WT (n = 20), dma-1(cbc1) (n = 18), and dma-1; klp-6p::dma-1 (n = 20). Replicate #3, WT (n = 22), *dma-1(cbc1)* (n = 21), and *dma-1; klp-6p::dma-1 (n = 23)*. (C) Expression of *sax-7* in the epidermis using the *dpy-7* promoter rescues the *sax-7* mutant branching phenotype. WT (n = 23), *sax-7* (n = 19), and *sax-7; dpy-7p::sax-7* (n = 19). (D) Expression of *mnr-1* in the epidermis using the *dpy-7* promoter rescues the *mnr-1* mutant branching phenotype. Replicate #2, WT (n = 19), *mnr-1* (n = 21), and *mnr-1; dpy-7p::mnr-1* (n = 18). Replicate #3, WT (n = 19), *mnr-1* (n = 22), and *mnr-1; dpy-7p::mnr-1* (n = 20). Data was analyzed with ANOVA followed by Tukey’s test for multiple comparisons to determine significance (B-D). ***p ≤0.001 and ****p ≤0.0001. Error bars are the standard error of the mean. (E) Overexpression of DMA-1 in the IL2s using the *klp-6* promoter does not induce ectopic branching during adult; WT (n = 10) and *klp-6p::dma-1* (n = 17). Representative, epifluorescent images of unbranched adult IL2 dendrites, wild type (top) and DMA-1 overexpression (bottom). IL2 neurons are visualized by *klp-6p::gfp* (A and E). Scale bars, 10 μm.
(TIF)

S2 Fig IL2 and FLP branching following L1 arrest. (A) Epifluorescent micrographs of dauers showing the IL2 body wall branches. Dauers isolated from pheromone plates not having gone through L1 arrest (top) and dauers that arrested at L1 and then grew to dauer in the presence of pheromone (bottom). We observed no noticeable differences in branching regardless of the L1 arrest. IL2 neurons are labeled with *klp-6p*::*gfp*. (B) Epifluorescent micrographs of adults showing body wall branches. Adults grown on replete food (top) and adults following L1 arrest (bottom). We observed no noticeable differences in branching regardless of the L1 arrest. FLP arbors (red arrows) and ALM neurite (yellow arrow). FLP neurons are labeled with *mec-7p*::*gfp*. Scale bars, 10μm.
(TIF)

S3 Fig Effect of membrane trafficking and the unfolded protein response on IL2 arborization. (A) Mutation in *rab-10* (n = 16) does not affect the number of IL2 body wall branches. The *exoc-8* mutant (n = 21) has a slight increase in the number of body wall branches compared to wild-type (n = 17). (B) Quantification of IL2 arborization in *rab-10* paralogs. Mutations in *rab-8* (n = 19) and *rab-35* (n = 21) do not affect IL2 branch number compared to wild-type (n = 22). (C) Quantification of IL2 arborization in unfolded protein response mutants. *xbp-1* (n = 19) has a slight increase in branch number, while *skr-5* (n = 21), *F47F2*.*1* (n = 21), and *tps-1* (n = 21) do not differ from wild-type in the number of body wall branches (n = 18). (D) Localization of *dma-1p*::*dma-1*::*gfp* in the *rab-10* and *ire-1* mutants. In the wild type (left), *rab-10* mutant (middle), and *ire-1* mutant dauers (right), *dma-1p*::*dma-1*::*gfp* is localized to the IL2 dendrite (red arrows). For statistical comparisons between more than two groups (A and C), we used ANOVA followed by Tukey’s test for multiple comparisons to determine significance. For statistical comparisons between two groups (B and C) we used t-tests to determine statistical significance. “ns” p >0.05, *p ≤0.05, **p ≤0.01. Error bars are the standard error of the mean. Scale bars, 10μm.
(TIF)

S4 Fig The role of dauer formation genes in FLP arborization and dauer-specific tissue remodeling. (A) Z-projection confocal micrograph of *daf-16(m27); daf-7(e1372)* adult FLP neurons shows a fully arborized FLP dendrite. (A’) Schematic of *daf-16(m27); daf-7(e1372)* FLP dendrites. (B) Z-projection confocal micrograph lateral view of a *daf-18(e1375); daf-7(e1372)* adult with extensive FLP arbor. (B’) Schematic of *daf-18(e1375); daf-7(e1372)* FLP dendrites. (C) Z-projection confocal micrograph of FLP neurons in *daf-9(m540)* mutant adults. (C’) Schematic of *daf-9(m540)* FLP dendrites. FLP neurons are visualized by *mec-7p*::*gfp*. FLP cell body (red asterisks), ALM/AVM neuronal processes (yellow arrows). Scale bars, 10 μm.
(TIF)

S1 Table List of *C*. *elegans* strains used in this study. (XLSX)

S2 Table List of plasmids and associated primers. (XLSX)
