# Supplementary figures and images for: Mutually exclusive dendritic arbors in C. elegans neurons share a common architecture and convergent molecular cues
Source: PLoS Genet. 2020 Sep 30;16(9):e1009029. doi: 10.1371/journal.pgen.1009029 (PMC7549815; doi:10.1371/journal.pgen.1009029)

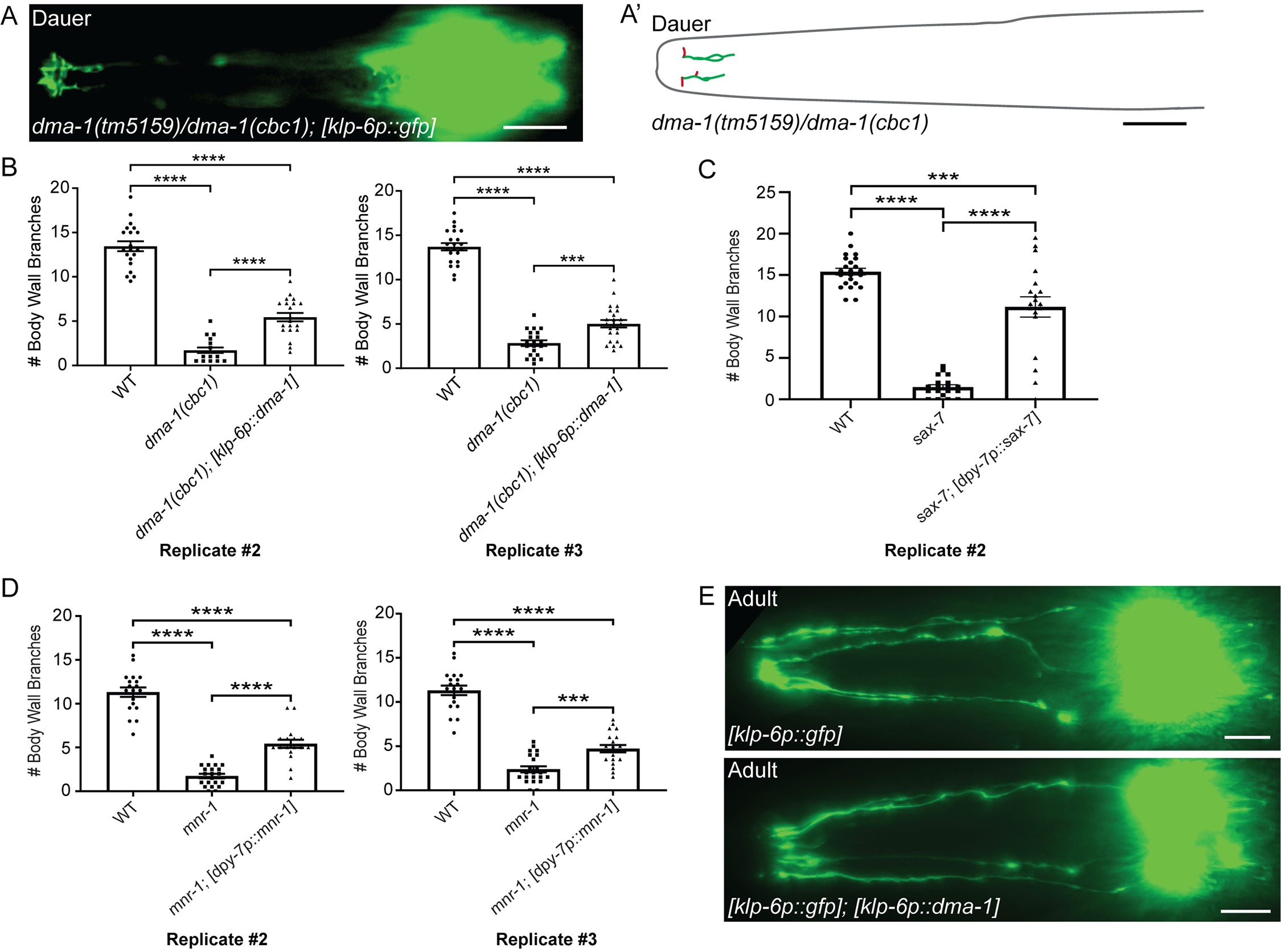

Supplement: S1 Fig — (A) dma-1(tm5159)/dma-1(cbc1) heterozygotes show a reduction of branches. (A’) Schematic drawings traced from the accompanying micrographs indicate the IL2 dendrites that are visible in the images. Dorsal midline branches (green) and body wall branches (red). (B) Quantification of IL2-specific DMA-1 rescue. Replicate #2, WT (n = 20), dma-1(cbc1) (n = 18), and dma-1; klp-6p::dma-1 (n = 20). Replicate #3, WT (n = 22), dma-1(cbc1) (n = 21), and dma-1; klp-6p::dma-1 (n = 23). (C) Expression of sax-7 in the epidermis using the dpy-7 promoter rescues the sax-7 mutant branching phenotype. WT (n = 23), sax-7 (n = 19), and sax-7; dpy-7p::sax-7 (n = 19). (D) Expression of mnr-1 in the epidermis using the dpy-7 promoter rescues the mnr-1 mutant branching phenotype. Replicate #2, WT (n = 19), mnr-1 (n = 21), and mnr-1; dpy-7p::mnr-1 (n = 18). Replicate #3, WT (n = 19), mnr-1 (n = 22), and mnr-1; dpy-7p::mnr-1 (n = 20). Data was analyzed with ANOVA followed by Tukey’s test for multiple comparisons to determine significance (B-D). ***p ≤0.001 and ****p ≤0.0001. Error bars are the standard error of the mean. (E) Overexpression of DMA-1 in the IL2s using the klp-6 promoter does not induce ectopic branching during adult; WT (n = 10) and klp-6p::dma-1 (n = 17). Representative, epifluorescent images of unbranched adult IL2 dendrites, wild type (top) and DMA-1 overexpression (bottom). IL2 neurons are visualized by klp-6p::gfp (A and E). Scale bars, 10 μm. (TIF) [file pgen.1009029.s001.tif]

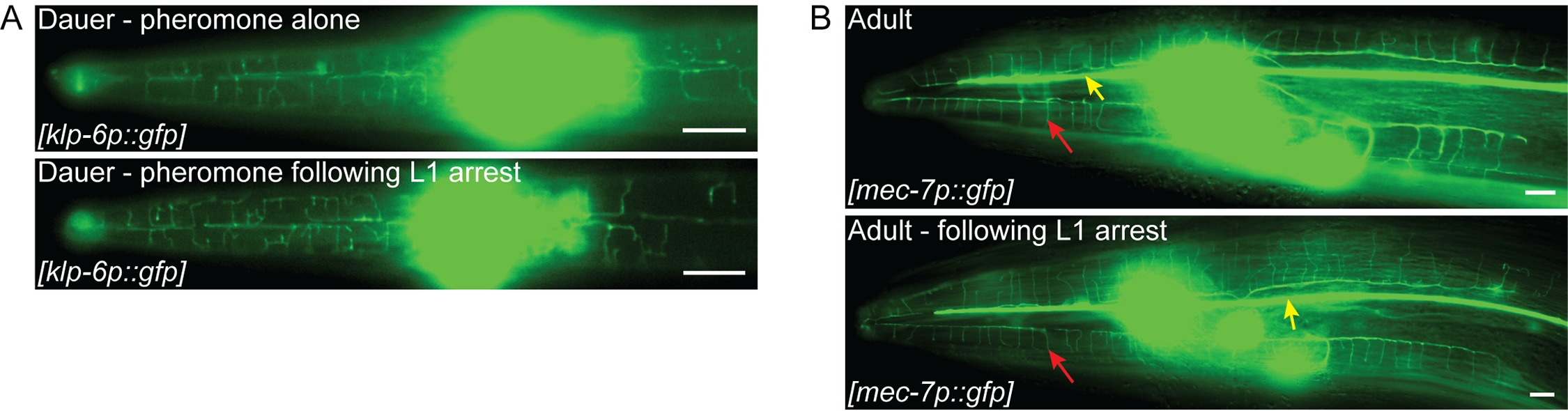

Supplement: S2 Fig — (A) Epifluorescent micrographs of dauers showing the IL2 body wall branches. Dauers isolated from pheromone plates not having gone through L1 arrest (top) and dauers that arrested at L1 and then grew to dauer in the presence of pheromone (bottom). We observed no noticeable differences in branching regardless of the L1 arrest. IL2 neurons are labeled with klp-6p::gfp. (B) Epifluorescent micrographs of adults showing body wall branches. Adults grown on replete food (top) and adults following L1 arrest (bottom). We observed no noticeable differences in branching regardless of the L1 arrest. FLP arbors (red arrows) and ALM neurite (yellow arrow). FLP neurons are labeled with mec-7p::gfp. Scale bars, 10μm. (TIF) [file pgen.1009029.s002.tif]

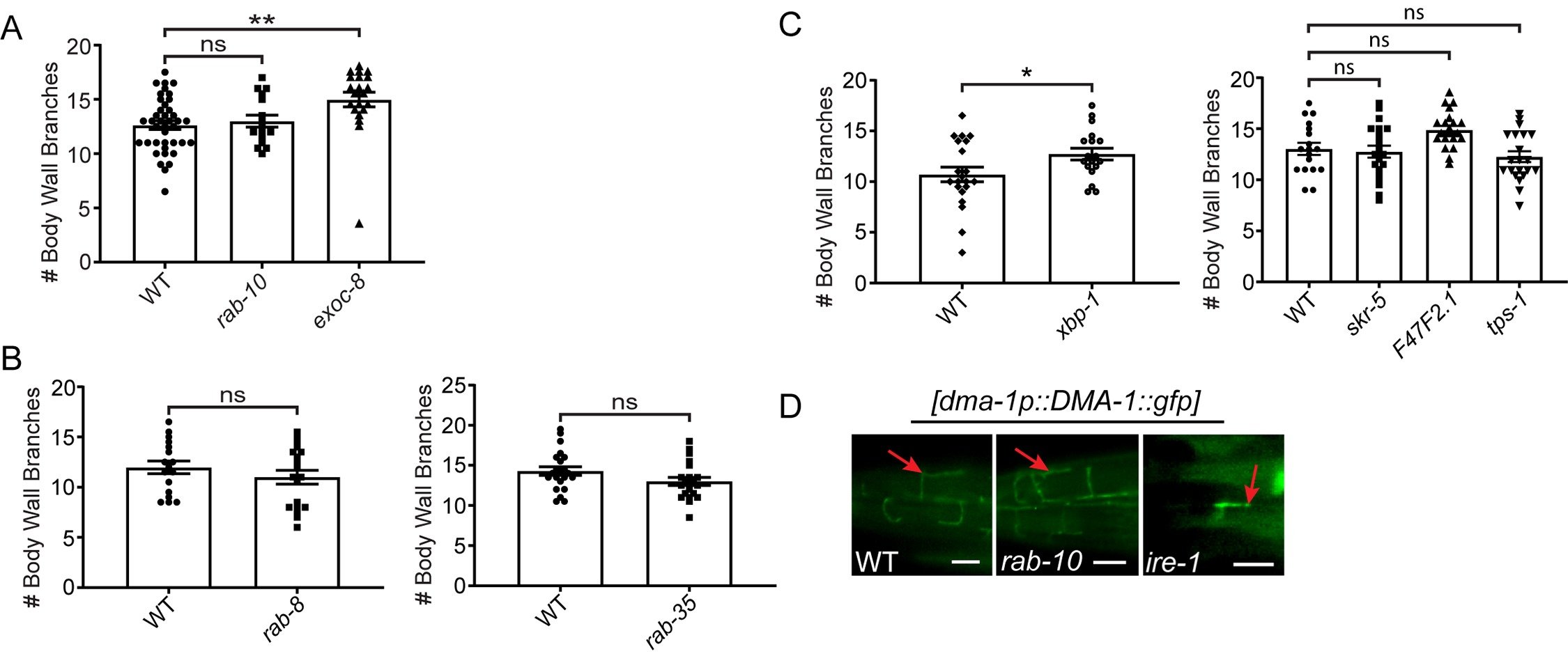

Supplement: S3 Fig — (A) Mutation in rab-10 (n = 16) does not affect the number of IL2 body wall branches. The exoc-8 mutant (n = 21) has a slight increase in the number of body wall branches compared to wild-type (n = 17). (B) Quantification of IL2 arborization in rab-10 paralogs. Mutations in rab-8 (n = 19) and rab-35 (n = 21) do not affect IL2 branch number compared to wild-type (n = 22). (C) Quantification of IL2 arborization in unfolded protein response mutants. xbp-1 (n = 19) has a slight increase in branch number, while skr-5 (n = 21), F47F2.1 (n = 21), and tps-1 (n = 21) do not differ from wild-type in the number of body wall branches (n = 18). (D) Localization of dma-1p::dma-1::gfp in the rab-10 and ire-1 mutants. In the wild type (left), rab-10 mutant (middle), and ire-1 mutant dauers (right), dma-1p::dma-1::gfp is localized to the IL2 dendrite (red arrows). For statistical comparisons between more than two groups (A and C), we used ANOVA followed by Tukey’s test for multiple comparisons to determine significance. For statistical comparisons between two groups (B and C) we used t-tests to determine statistical significance. “ns” p >0.05, *p ≤0.05, **p ≤0.01. Error bars are the standard error of the mean. Scale bars, 10μm. (TIF) [file pgen.1009029.s003.tif]

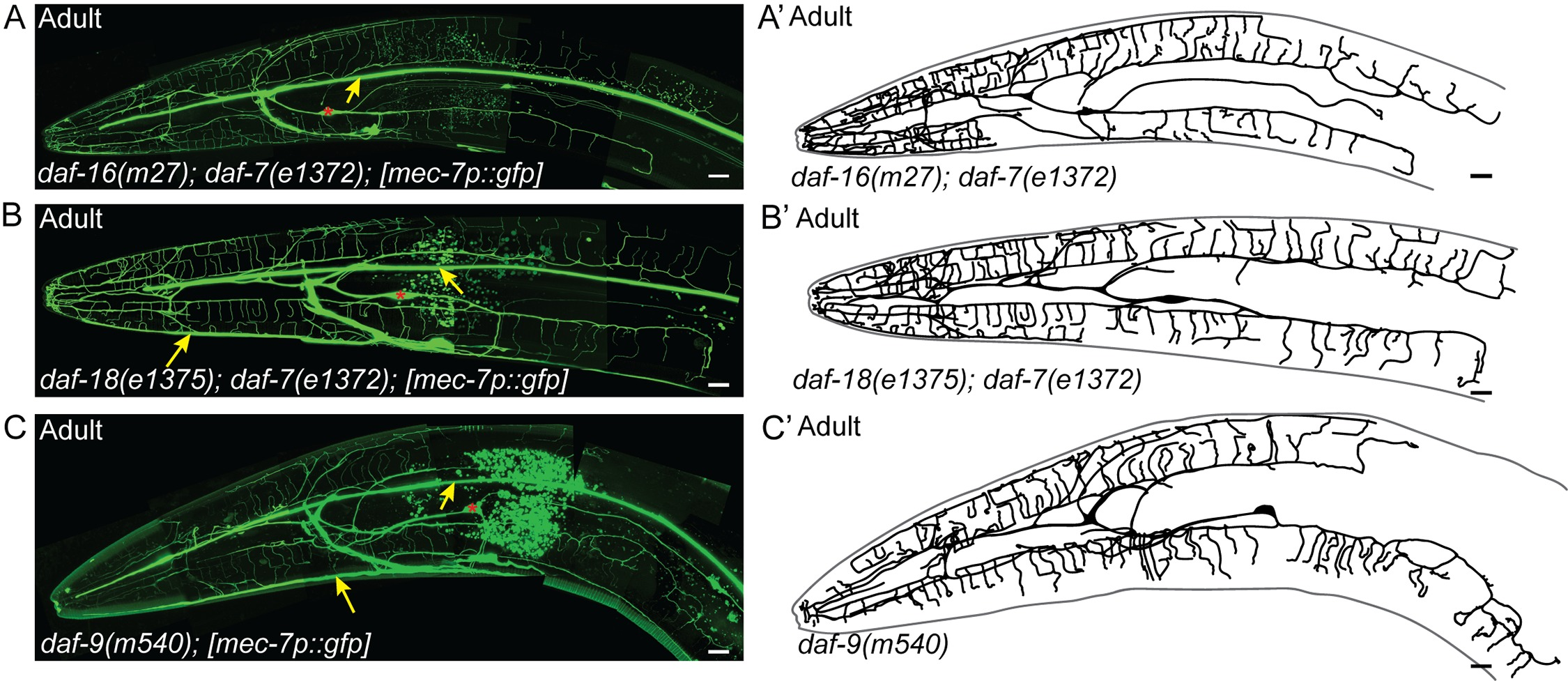

Supplement: S4 Fig — (A) Z-projection confocal micrograph of daf-16(m27); daf-7(e1372) adult FLP neurons shows a fully arborized FLP dendrite. (A’) Schematic of daf-16(m27); daf-7(e1372) FLP dendrites. (B) Z-projection confocal micrograph lateral view of a daf-18(e1375); daf-7(e1372) adult with extensive FLP arbor. (B’) Schematic of daf-18(e1375); daf-7(e1372) FLP dendrites. (C) Z-projection confocal micrograph of FLP neurons in daf-9(m540) mutant adults. (C’) Schematic of daf-9(m540) FLP dendrites. FLP neurons are visualized by mec-7p::gfp. FLP cell body (red asterisks), ALM/AVM neuronal processes (yellow arrows). Scale bars, 10 μm. (TIF) [file pgen.1009029.s004.tif]
